# The (In)visibility of Groomed Ski Runs

**DOI:** 10.1177/2041669519842895

**Published:** 2019-04-13

**Authors:** William J. Harrison

**Affiliations:** Queensland Brain Institute, The University of Queensland, Brisbane, Australia

**Keywords:** contrast sensitivity, first tracks, groomed snow, spatial vision

## Abstract

I analyse the visibility of “groomed” ski runs under different lighting conditions. A model of human contrast sensitivity predicts that the spatial period of groomed snow may render it invisible in the shade or on overcast days. I confirm this prediction with visual demonstrations and make a suggestion to improve visibility.

Moments before I “stacked it”^1^ while snowboarding on Cougar Alley, Big White Ski Resort (Canada), I appreciated that the blue run was as much a challenge for my contrast sensitivity as it was for my aching thighs. Indeed, poor visibility significantly raises the risk of injury during snow sports (Hume, Lorimer, Griffiths, Carlson, & Lamont, 2015). I attempted the ski trail in such highly overcast conditions that no shadows were visible, and, more importantly, visual features indicating bumps, hollows, and moguls were often of such low contrast that they were effectively invisible. The trail was freshly groomed, a machine process that involves compacting and smoothing the snow. Such grooming is thought to improve skier and snowboarder safety (Bergstrom, 2004). As shown in Figure 1(a), grooming results in a grating-like appearance of the snow, particularly when sunlit. The grating orientation approximates the slope of the hill such that, under well-lit conditions, grooming provides the skier or snowboarder with important visual cues as to changes in the gradient of the slope. I thus wondered how grooming may facilitate or impair visibility of the snow under different lighting conditions.

**Figure 1.**
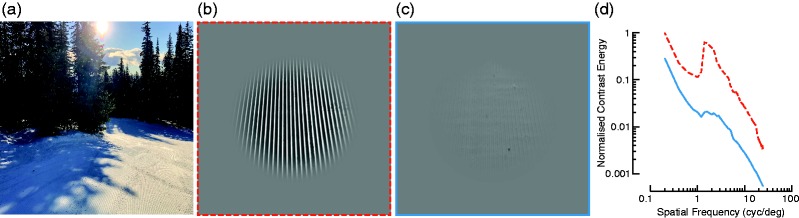
(a) A groomed ski run. A patch of groomed snow in sunlight (b) and in shade (c) and their image spectra (d). Photos in (b) and (c) were taken minutes apart on the same slope and day with an iPhone X. Images were converted to greyscale, assumed to have an encoding gamma of 2, scaled to be in the range [−1 1], and normalized so that the mean pixel intensity was zero. Finally, I applied a circular aperture with a cosine edge to minimize wrapping artifacts during analysis. (d) The dashed red line and solid blue line show normalized contrast energy for the sunlit and shaded patch, respectively, as a function of spatial frequency. Contrast energy was calculated in the frequency domain, summing across orientations and normalized to the maximum contrast across images.

I first asked how well the amplitude spectrum of the groomed snow aligns with the human contrast sensitivity function. Figure 1(b) and (c) shows the photos I took on a clear, sunny morning of freshly groomed snow in sunlight and in the shade of trees, respectively. The centre of each image is approximately 90 cm from my feet, and the camera was at a height of approximately 178 cm. The spatial period in the centre of the groomed snow is therefore 1.38 cycles per degree of visual angle (cyc/deg; one cycle per 2.53 cm). Figure 1(d) shows the contrast energy of each image, normalised such that the peak contrast is equal to one. There is a roughly linear relationship between contrast and spatial frequency (“1/f”) but with notable bumps at approximately 1.5 cyc/deg. The high contrast of groomed snow at 1.5 cyc/deg corresponds well with the peak human sensitivity to grating stimuli (e.g., Watson & Ahumada, 2005) and natural images (Bex & Makous, 2002). I could not establish whether this close match between the spatial frequency of groomed snow and peak contrast sensitivity was coincidental or by design: Patents only describe an operator’s ability to see outside the grooming vehicle but not the visibility of the groomed snow itself (e.g., Brandt, 1989). However, this coincidence only holds for the specific viewing distance measured here, a point to which I will return below. Regardless, between 1 and 10 cyc/deg, the shaded snow had on average 8.8 times less contrast energy than the sunlit snow.

I used a model of contrast sensitivity of human foveal vision (Watson & Ahumada, 2005) to predict the visibility of groomed snow in the sun and shade. These predictions are shown in Figure 2(a) for patches of snow filtered into different spatial frequency bands (see Figure 2 caption). The model predicts that sunlit snow is visible at all spatial scales (filled red data), but that shaded snow is only visible within the midrange frequencies (filled blue data). I tested these model predictions with the visual demonstration shown in Figure 2(b) in which the shaded and sunlit snow patches are filtered into the same spatial frequency bands as measured in Figure 2(a). Visual inspection of this demonstration reveals that the model predictions are highly accurate.

**Figure 2.**
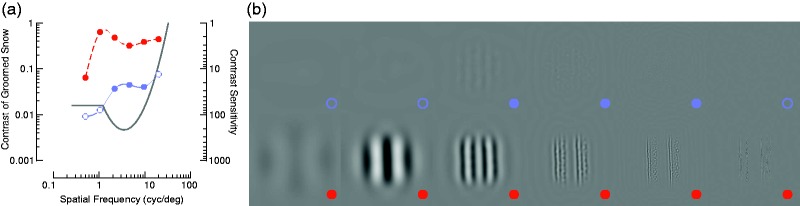
Prediction of the visibility of groomed snow based on a model of the human contrast sensitivity function. (a) The red and blue data, plotted on the left ordinate, are the maximum Weber contrast values calculated across 4 cyc/deg patches of sunlit and shaded snow, respectively, in separable and equally spaced frequency bands. The solid line shows a truncated log-parabolic model of contrast sensitivity, plotted on the right ordinate, with parameters as described by Watson and Ahumada (2005). Contrast above the solid line should be visible, whereas contrast below the solid line should be invisible, as indicated by the filled and open points, respectively. (b) A demonstration of the change in visibility of groomed snow as a nonlinear function of spatial frequency and contrast. The figure should be viewed from a distance such that one cycle subtends one degree of visual angle. The top and bottom rows show shaded and sunlit snow patches, respectively, with spatial frequency increasing from left to right. The predictions from (a) are shown in the bottom right of each patch and correspond very well with the subjective visibility of each patch.

The nonlinear interaction between contrast sensitivity and spatial frequency is critical for estimating visibility in low contrast regions of a groomed ski slope in the real world. At a distance of approximately 10 m, the model predicts that groomed snow becomes close to invisible at all spatial scales when in the shade. This prediction can be confirmed by viewing Figure 2(b) from a large distance.

To improve visibility of groomed snow at a range of viewing distances, a simple^2^ modification to snow grooming may suffice. Consider that, as viewing distance increases, the shaded, groomed snow becomes less visible because its spatial period on the retinae approaches the upper frequency limits of the contrast sensitivity function. Therefore, a second, lower periodicity groom should improve visibility in equivalently low contrast conditions. A “hybrid” groom that combines periodicities would remain visible at a broader range of viewing distances than groomed snow with only a single peak frequency. I simulate such a hybrid groom pattern in Figure 3. Figure 3(a) and (b) shows high and low spatial scales, respectively. Figure 3(c) shows the result of combining these components, and Figure 3(d) demonstrates the superior visibility of the hybrid groom at a simulated greater viewing distance.

**Figure 3.**
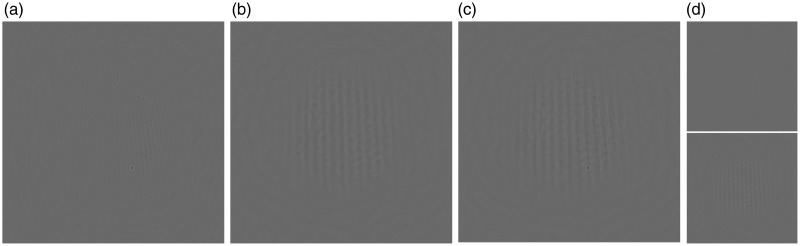
The visibility of groomed snow in low contrast conditions may be improved by combining grooms of different periodicities. (a) The same shaded snow patch as in Figure 1(c). (b) The same patch again but enlarged such that its periodicity is four times lower than in (a). (c) The simulated hybrid groom, which combines the periodicity of (a) and (b). This groom pattern should be visible across a greater range of viewing distances than either component alone. (d) Improved visibility of the hybrid groom is demonstrated after scaling the original periodicity (top panel) and the hybrid groom (bottom panel) by 50%, equivalent to doubling the viewing distance. Whereas the groomed snow is rendered invisible in the top panel, it is still visible in the bottom panel, owing to the second, lower frequency groom period. Prior to combining, snow patches in (a) and (b) were spatially filtered such that their contrast energies are contained within independent frequency bands.

The data presented here provide a preliminary analysis of the (in)visibility of groomed snow. My results reveal that a groom pattern may render the snow invisible in low light conditions at viewing distances relevant in snow sports. In particularly low visibility conditions, I noticed I had significant problems with correctly perceiving the depth of the snow due to errors in binocular fusion, vergence, and accommodation. Future analyses could also consider other factors contributing to visibility of groomed snow, such as how sensitivity varies in the visual periphery, with yellow-tinted lenses (e.g., Kinney, Luria, Schlichting, & Neri, 1983), during eye, body and head movements, and how different atmospheric conditions affect groom contrast. Nonetheless, my suggested hybrid groom pattern requires only minor modification to the present groom design and could improve visibility – and therefore safety – in poor lighting conditions. I am hopeful a funding agency will give me the opportunity to explore remaining questions next winter.

## Notes

1 “To stack it” is Australian slang synonymous with “to go A-over-T,” or, simply, “to crash.”

2 I write “simple” to refer to the required change to the visual design of the groomed snow. I obviously have no idea how hard it is to modify the grooming machinery to achieve this.

## References

[bibr1-2041669519842895] Bergstrom K. A. (2004) Effect of trail design and grooming on the incidence of injuries at alpine ski areas. British Journal of Sports Medicine 38: 264–268. doi:10.1136/bjsm.2002.000270. 15155423 10.1136/bjsm.2002.000270 PMC1724808

[bibr2-2041669519842895] Bex P. J. Makous W. (2002) Spatial frequency, phase, and the contrast of natural images. Journal of the Optical Society of America A 19: 1096–1106. doi:10.1364/JOSAA.19.001096. 10.1364/josaa.19.001096 12049346

[bibr3-2041669519842895] Brandt, C. R. (1989). *US4860465A*. *United States*. Retrieved from https://patents.google.com/patent/US4860465A/en.

[bibr4-2041669519842895] Hume P. A. Lorimer A. V. Griffiths P. C. Carlson I. Lamont M. (2015) Recreational snow-sports injury risk factors and countermeasures: A meta-analysis review and Haddon matrix evaluation. Sports Medicine 45: 1175–1190. doi:10.1007/s40279-015-0334-7. 25946993 10.1007/s40279-015-0334-7

[bibr5-2041669519842895] Kinney J. A. S. Luria S. M. Schlichting C. L. Neri D. F. (1983) The perception of depth contours with yellow goggles. Perception 12: 363–366. doi:10.1068/p120363. 6669463 10.1068/p120363

[bibr6-2041669519842895] Watson A. B. Ahumada A. J. (2005) A standard model for foveal detection of spatial contrast. Journal of Vision 5: 6 doi:10.1167/5.9.6. 10.1167/5.9.6 16356081

